# Projected Outcomes of Optimized Statin and Ezetimibe Therapy in US Military Veterans with Coronary Artery Disease

**DOI:** 10.1001/jamanetworkopen.2023.29066

**Published:** 2023-08-28

**Authors:** Christopher P. Kovach, Elise C. Mesenbring, Prerna Gupta, Thomas J. Glorioso, P. Michael Ho, Stephen W. Waldo, Gregory G. Schwartz

**Affiliations:** 1 Division of Cardiology, Department of Medicine, University of Washington, Seattle; 2 Division of Cardiology, Department of Medicine, University of Colorado, Aurora; 3 Rocky Mountain Regional VA Medical Center, Aurora, Colorado; 4 Denver Research Institute, Aurora, Colorado; 5 Clinical Assessment, Reporting, and Tracking Program, Office of Quality and Patient Safety, Veterans Health Administration, Washington, DC

## Abstract

**Importance:**

Many patients with coronary artery disease (CAD) do not achieve the guideline-directed goals for low-density lipoprotein cholesterol (LDL-C) levels.

**Objective:**

To estimate reductions in the rates of adverse events associated with CAD in a large US military veteran population that may be achieved through use of optimized statin therapy alone or with ezetimibe compared with the prevailing lipid-lowering therapy (LLT).

**Design, Setting, and Participants:**

In this observational cohort study, US military veterans with CAD were identified by coronary angiography between June 2015 and September 2020 across 82 US Department of Veterans Affairs health care facilities.

**Exposures:**

The exposures were observed LLT, LLT with an optimized statin regimen, and LLT with optimized statin and ezetimibe.

**Main Outcomes and Measures:**

Observed rates of death, myocardial infarction, stroke, and coronary revascularization, and potential reductions in those outcomes with optimized LLT based on expected further reductions in LDL-C levels and application of formulas from The Cholesterol Treatment Trialists’ Collaboration.

**Results:**

The analysis cohort comprised 111 954 veterans (mean [SD] age, 68.4 [8.8] years; 109 390 men [97.7%]; 91 589 White patients [81.8%]; 17 592 Black patients [15.7%]). The median (IQR) observation period for this study was 3.4 (2.1-4.0) years. At the time of index angiography, 66 877 patients (59.7%) were treated with statin therapy, and 623 patients (0.6%) were treated with ezetimibe. At 6 months, the number of patients with statin prescriptions increased to 74 400 (68.7%), but the number of patients with high-intensity statin prescriptions was only 57 297 (52.9%). At 6 months, ezetimibe use remained low (n = 1168 [1.1%]), and LDL-C levels were 70 mg/dL or more in 56 405 patients (52.1%). At 4 years, observed incidences of death, myocardial infarction, stroke, and coronary revascularization were 21.6% (95% CI, 21.3%-21.8%), 5.0% (95% CI, 4.9%-5.2%), 2.2% (95% CI, 2.1%-2.3%), and 15.4% (95% CI, 15.2%-15.7%), respectively. With optimized statin treatment, projected absolute reductions in these incidences were 1.3% (95% CI, 0.9%-1.7%), 0.8% (95% CI, 0.7%-1.0%), 0.2% (95% CI, 0.1%-0.3%), and 2.3% (95% CI, 2.0%-2.7%), respectively. With optimized statin and ezetimibe treatment, projected absolute reductions were 1.8% (95% CI, 1.2%-2.4%), 1.1% (95% CI, 0.9%-1.3%), 0.3% (95% CI, 0.2%-0.4%), and 3.1% (95% CI, 2.6%-3.6%), respectively.

**Conclusions and Relevance:**

In this cohort study of veterans with CAD, suboptimal LLT was prevalent in the clinical setting. Optimization of statin therapy was projected to produce clinically relevant reductions in the risks of death and cardiovascular events. Despite a lesser lipid-lowering efficacy of ezetimibe, its widespread use on a population level in conjunction with optimized statin therapy may be associated with further meaningful reductions in cardiovascular risk.

## Key Points

**Question:**

What are the potential unrealized opportunities to reduce adverse cardiovascular events among a US military veteran population with coronary artery disease using optimized oral lipid-lowering therapy?

**Findings:**

In this cohort study of 111 954 US military veterans with coronary artery disease documented during an index coronary angiography, lipid-lowering therapy in clinical practice was frequently suboptimal. Optimizing treatment with a high-intensity statin regimen was estimated to reduce the risk of death and cardiovascular events in a clinically meaningful way, with adjunctive use of ezetimibe adding notably to those projected benefits.

**Meaning:**

Substantial opportunities may exist in this population to further reduce cardiovascular risk through optimized use of both the highest-intensity clinically appropriate statin regimen and ezetimibe.

## Teaser

This cohort study estimates the projected reductions in rates of adverse events associated with coronary artery disease in a large US military veteran population when clinicians optimize oral lipid-lowering therapy as clinically appropriate.

## Introduction

Despite international guidelines for low-density lipoprotein cholesterol (LDL-C) reduction among patients with atherosclerotic cardiovascular disease (ASCVD), attainment of LDL-C goals often remains suboptimal in clinical practice.^1,2,3^ Consequently, opportunities to reduce the risk of death and ASCVD events remain partially unfulfilled. A meta-analysis by the Cholesterol Treatment Trialists’ (CTT) Collaboration^4^ of randomized clinical trials with statins demonstrated a consistent association between absolute reduction in LDL-C concentration and relative reduction in risk of death and major vascular events. Data from trials with ezetimibe appear to support this relationship with reasonable fidelity.^4,5,6^ Treatment with a high-intensity statin regimen results in an approximate 50% LDL-C reduction, while addition of ezetimibe provides a further 13% to 18% reduction in LDL-C.^5,7,8^ Ezetimibe is recommended as the first nonstatin agent to prescribe when LDL-C goals are not attained with an optimized statin regimen alone.^1,2^ However, despite ezetimibe’s safety, tolerability, and low cost, its use remains less than 10% in clinical trial populations.^9,10,11^ Optimizing lipid-lowering therapy (LLT) with both statin and ezetimibe treatment therefore offers an efficient strategy to achieve substantial LDL-C reduction and consequent clinical benefit.^12,13,14,15,16^

We leveraged data from the US Department of Veterans Affairs (VA) health care system to assess the prevalence of LLT and attainment of LDL-C goals in patients with angiographic coronary artery disease (CAD). We also sought to quantify the unrealized opportunity to improve clinical outcomes with optimized statin and ezetimibe therapy. We hypothesize that underutilization of both statins and ezetimibe is responsible for substantial unrealized clinical benefit of LLT.

## Methods

### Population

In this cohort study of US military veterans, data from the VA Clinical Assessment Reporting and Tracking Program were used to identify all patients aged at least 18 years who underwent coronary angiography demonstrating obstructive or nonobstructive CAD from June 1, 2015, to September 30, 2020. Inclusion and exclusion criteria are described in the eMethods in Supplement 1. The analysis was approved by the Colorado Multiple Institutional Review Board, and because data were deidentified, informed consent was waived. The study followed the Strengthening the Reporting of Observational Studies in Epidemiology (STROBE) reporting guideline for cohort studies.^17^

### Baseline LDL-C and LLT

Baseline LDL-C was established as the measurement in closest temporal proximity to angiography, considering all results 12 months prior (preferred) or 3 months following the procedure (if no preprocedure LDL-C measurement was available). Statin and/or ezetimibe treatment was considered to be active beginning on the 14th day after the release of the prescription to the patient (to account for time to receive the prescription and to achieve steady state LDL-C on treatment) and was considered to remain active for the number of days prescribed plus 14 days. If active LLT status did not change between the date of the nearest LDL-C measurement and the date of the angiography, that LLT and the nearest LDL-C measurement were considered baseline. If active LLT changed between the date of LDL-C measurement and index angiography, baseline LDL-C levels were imputed using the change in LDL-C anticipated from the change in therapy (eTable 1 in Supplement 1).

### Observed LDL-C Trajectory and Prescribed LLT

For each patient, LDL-C levels and LLT during the observation period were determined using all available LDL-C and prescription data. Levels of LDL-C were assumed to remain unchanged from the prior measurement until either a new LDL-C measurement was acquired or a change in active LLT was recorded. Each LDL-C measurement was associated with concurrent active LLT as defined previously. The LDL-C values were imputed when the active LLT changed from the regimen associated with the most recent measurement using the expected change in LDL-C with the new regimen (eTable 1 in Supplement 1). Imputed LDL-C values were superseded by the next documented LDL-C measurement.

### Projected LDL-C With Optimized LLT

During the observation period, time-varying optimal LDL-C levels were projected for each patient as the observed LDL-C value minus the expected reduction in LDL-C with clinically appropriate optimized statin therapy alone or optimized statin therapy with ezetimibe (eTable 1 in Supplement 1). Optimized statin therapy was individualized (reduced dose or no statin) for patients with relative or absolute contraindications to treatment (eMethods in Supplement 1). For patients without contraindications, optimized statin therapy was considered high-intensity treatment with atorvastatin 80 mg daily, assumed to result in a 54% reduction in LDL-C from an untreated baseline. Treatment with ezetimibe 10-mg was considered an appropriate addition in all cases. Ezetimibe treatment was assumed to result in a further 15% reduction in LDL-C levels from the LDL-C level on an optimized statin regimen.^8^

### Outcomes

The outcomes evaluated were all-cause death, rehospitalization for myocardial infarction, rehospitalization for stroke, and coronary revascularization more than 30 days after the index angiography. All patients had a minimum follow-up period of 30 days and were followed up to death, 4 years, or September 30, 2021, whichever came first.

### Statistical Analysis

Standard inferential statistics were used to assess differences between groups. Weighted averages of each patient’s LDL-C levels with observed LLT, optimized statin therapy, and optimized statin therapy with ezetimibe added were calculated in 30-day intervals for the observation period. Linear mixed models with a random intercept for the patient were created across 1000 bootstrap samples to model average LDL-C levels for each treatment scenario. A natural spline with 6 degrees of freedom modeled the association between days following the index angiography and the LDL-C value reflecting each treatment scenario. Within each bootstrap sample at every 6-month time point, the observed cumulative incidence of each clinical outcome was determined, and a rate ratio (RR) per each 38.67 mg/dL (to convert to millimoles per liter, multiply by 0.0259) LDL-C reduction was simulated from a lognormal distribution for each outcome based on the empirical associations derived in the CTT analyses for each outcome of interest.^4^ This formula:
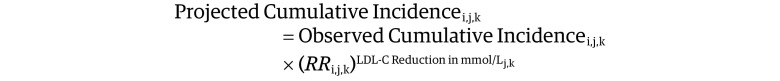
was then used to calculate the projected cumulative event incidence using the expected reduction in LDL-C levels with optimization of LLT and the observed cumulative incidence for the outcome *i* at time point *j* in bootstrap model *k* using the CTT associations (RR*i*, *j*, *k*; RR per 1 mmol/L reduction in LDL-C levels; eMethods in Supplement 1). The estimated reduction in LDL-C levels and cumulative incidence for each outcome–time point combination was calculated from the average bootstrap results with percentiles 2.5 and 97.5 of the distribution representing the 95% CI. Estimates of the number needed to treat to prevent 1 event were calculated for each outcome. Secondary analyses using similar methods compared patients who presented with acute coronary syndrome (ACS) with those who did not at the time of index angiography.

Statistical, descriptive, and graphical analyses were performed with R statistical software, version 3.5.3 (R Project for Statistical Computing) using packages splines and cmprsk.^18^ A 2-sided *P* value of <.05 was considered statistically significant.

## Results

### Population

A total of 145 722 patients who underwent coronary angiography at 82 VA health care facilities were found to have CAD during the study period. Of these, 10 204 patients were excluded for insufficient data to determine baseline LDL-C levels, 14 327 for nonstandard statin regimens, 2144 for treatment with a proprotein convertase subtilisin/kexin type 9 (PCSK9) inhibitor, 4665 for dialysis dependence, 3840 for estimated glomerular filtration rate of less than 15 mL/min, 22 for being less than 18 years old, and 1836 for death within 30 days of index angiography; reasons were not mutually exclusive. The final analytic cohort comprised 111 954 patients (mean [SD] age, 68.4 [8.8] years; 109 390 men [97.7%]; 91 589 [81.8%] White and 17 592 [15.7%] Black patients; Hispanic patients [n = 5941] comprised 5.3% of the cohort). Because patients from historically marginalized groups may be at risk of poorer medical outcomes, race and ethnicity were determined to open inquiry into measuring and potentially improving those outcomes (eFigure 1 in Supplement 1). The median (IQR) follow-up period for all patients was 3.4 (2.1-4.0) years.

### Baseline Characteristics

Baseline characteristics of the overall cohort and separately for patients presenting with or without ACS are shown in Table 1. A total of 30 037 patients (26.8%) presented with ACS. Patients with ACS had more comorbidities, higher rates of P2Y12 inhibitor use, and lower rates of β-blocker, calcium channel blocker, and renin-angiotensin system antagonist use compared with patients without ACS. Patients with ACS had more complex coronary anatomy compared with those without ACS based on the VA SYNTAX score (Veterans Affairs Synergy Between Percutaneous Coronary Intervention With Taxus and Cardiac Surgery), a tool to help clinicians grade the complexity of CAD.^19,20^

**Table 1.** Baseline Characteristics of the Overall Cohort and for Patients Presenting With or Without ACS^a^

Characteristic	All patients, No. (%) (n = 111 954)	Non-ACS, No. (%) (n = 81 917)	ACS, No. (%) (n = 30 037)	*P* value^a^
Demographic				
Age, mean (SD), y	68.4 (8.8)	68.6 (8.5)	67.8 (9.5)	<.001
Sex				
Female	2564 (2.3)	1819 (2.2)	745 (2.5)	.01
Male	109 390 (97.7)	80 098 (97.8)	29 292 (97.5)	
Race				
White	91 589 (81.8)	67 519 (82.4)	24 070 (80.1)	<.001
Black	17 592 (15.7)	12 443 (15.2)	5149 (17.1)	
Other^b^	2773 (2.5)	1955 (2.4)	818 (2.7)	
Hispanic ethnicity	5941 (5.3)	3927 (4.8)	2014 (6.7)	<.001
Medical history				
CAD at index angiography				
Obstructive	76 162 (68.0)	51 933 (63.4)	24 229 (80.7)	<.001
Nonobstructive	35 792 (32.0)	29 984 (36.6)	5808 (19.3)	
VA SYNTAX, median (IQR)^c^	7 (2-17)	7 (1-16)	10 (4-19)	<.001
Prior MI/PCI/CABG	54 897 (49.0)	37 856 (46.2)	17 041 (56.7)	<.001
Prior MI	38 927 (34.8)	26 018 (31.8)	12 909 (43.0)	<.001
Prior PCI	36 233 (32.4)	24 656 (30.1)	11 577 (38.5)	<.001
Prior CABG	23 674 (21.1)	16 688 (20.4)	6986 (23.3)	<.001
Heart failure	35 741 (31.9)	26 916 (32.9)	8825 (29.4)	<.001
Prior stroke	11 347 (10.1)	7687 (9.4)	3660 (12.2)	<.001
Peripheral artery disease	24 606 (22.0)	17 560 (21.4)	7046 (23.5)	<.001
Diabetes	57 591 (51.4)	41 752 (51.0)	15 839 (52.7)	<.001
Chronic kidney disease	25 954 (23.2)	18 403 (22.5)	7551 (25.1)	<.001
Hypertension	102 267 (91.3)	74 730 (91.2)	27 537 (91.7)	.02
Hyperlipidemia	100 657 (89.9)	73 438 (89.6)	27 219 (90.6)	<.001
Atrial fibrillation	21 194 (18.9)	16 359 (20.0)	4835 (16.1)	<.001
COPD	30 365 (27.1)	22 015 (26.9)	8350 (27.8)	.002
Obesity^d^	55 177 (49.3)	41 252 (50.4)	13 925 (46.4)	<.001
Sleep apnea	37 916 (33.9)	28 096 (34.3)	9820 (32.7)	<.001
Tobacco use^e^	74 915 (66.9)	54 021 (65.9)	20 894 (69.6)	<.001
Alcohol use disorder	10 749 (9.6)	7306 (8.9)	3443 (11.5)	<.001
Other substance abuse	6163 (5.5)	4009 (4.9)	2154 (7.2)	<.001
Chronic HIV	720 (0.6)	481 (0.6)	239 (0.8)	<.001
Selected cardiovascular medications				
P2Y12 inhibitor	20 028 (17.9)	14 287 (17.4)	5741 (19.1)	<.001
β-Blocker	65 537 (58.5)	50 756 (62.0)	14 781 (49.2)	
CCB	29 313 (26.2)	21 776 (26.6)	7537 (25.1)	
ACEi/ARB/ARNI	60 269 (53.8)	45 762 (55.9)	14 507 (48.3)	
Indication for coronary angiography				
Acute coronary syndromes				
Unstable angina	12 680 (11.3)	0	12 680 (42.2)	<.001
NSTEMI	13 737 (12.3)	0	13 737 (45.7)	
STEMI	1740 (1.6)	0	1740 (5.8)	
Unspecified	1050 (0.9)	0	1050 (3.5)	
Chronic coronary syndromes				
Stable angina	24 026 (21.5)	23 718 (29.0)	308 (1.0)	<.001
Atypical chest pain	12 611 (11.3)	12 414 (15.2)	197 (0.7)	
Unspecified	5624 (5.0)	5614 (6.9)	10 (0.03)	
Heart failure	2349 (2.1)	2346 (2.9)	3 (0.01)	
Cardiomyopathy	3025 (2.7)	3021 (3.7)	4 (0.01)	
Valve disease	8228 (7.3)	8198 (10.0)	30 (0.1)	
Other^f^	17 467 (15.6)	17 189 (20.1)	278 (0.9)	
Missing data	9417 (8.4)	9417 (11.5)	0	

Abbreviations: ACEi, angiotensin-converting enzyme inhibitor; ACS, acute coronary syndrome; ARB, aldosterone receptor blocker; ARNI, angiotensin receptor-neprilysin inhibitor; CABG, coronary artery bypass grafting; CAD, coronary artery disease; CCB, calcium channel blocker; COPD, chronic obstructive pulmonary disease; HIV, human immunodeficiency virus; MI, myocardial infarction; NSTEMI, non–ST-elevation myocardial infarction; PCI, percutaneous coronary intervention; STEMI, ST-elevation myocardial infarction; VA SYNTAX, Veterans Affairs Synergy Between Percutaneous Coronary Intervention With Taxus and Cardiac Surgery.

^a^ *P* values reflect comparisons between non-ACS and ACS patient categories.

^b^ The category “other” for race indicates American Indian or Alaska Native, Asian, and Native Hawaiian or other Pacific Islander.

^c^ The VA SYNTAX score is a tool to help clinicians grade the complexity of CAD.

^d^ Obesity was defined by a body mass index, calculated as weight in kilograms divided by height in meters squared, of 30 or more.

^e^ Tobacco use includes prior or current tobacco use.

^f^ Other indications for coronary angiography include arrhythmia, asymptomatic ischemic, cardiogenic shock, tamponade, congenital heart disease, preoperative evaluation, pulmonary hypertension, syncope, transplant evaluation, and history of heart transplant.

**Figure 1.**
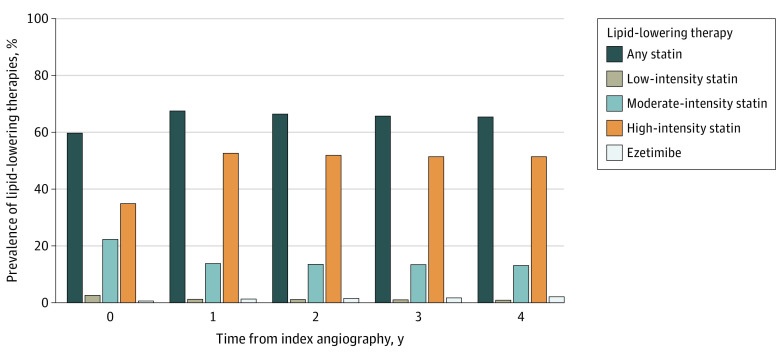
Prevalence of Lipid-Lowering Therapies During the Study Period At the time of index coronary angiography, 66 877 patients (59.7%) were prescribed a statin, 39 042 patients (34.9%) were prescribed a high-intensity statin regimen, and ezetimibe use was rare (623 [0.6%]). The proportion of patients prescribed a statin peaked at 3 months following index angiography and then remained generally stable from 6 months through 4 years after index angiography. The use of ezetimibe remained low throughout the study period.

At the time of index coronary angiography, 66 877 patients (59.7%) were prescribed a statin, and 39 042 patients (34.9%) were prescribed a high-intensity statin regimen (Figure 1; eTable 2 in Supplement 1). Ezetimibe use was rare (623 [0.6%]). Patients with an ACS presentation vs those without ACS were less likely to be prescribed a statin (52.0% vs 62.6%; *P* < .001) or a high-intensity statin (30.5% vs 36.5%; *P* < .001) at baseline. Baseline mean (SD) and median (IQR) LDL-C levels for the cohort were 79.7 (36.4) mg/dL and 74.0 (55.1-96.8) mg/dL, respectively, without significant differences between patients with ACS and without ACS. Baseline LDL-C levels were highest in patients who were treated with no statin and were sequentially lower among patients treated with low-intensity, moderate-intensity, and high-intensity statin therapy (eTable 3 in Supplement 1). Most patients (62 211 [55.6%]) had baseline LDL-C levels of 70 mg/dL or more.

### Postbaseline LLT, LDL-C Levels, and Outcomes

The median (IQR) time to first LDL-C measurement after angiography was 135 (57-267) days. At 6 months after index angiography, a postangiography measurement of LDL-C had been made in 66 001 (59.0%) of patients; at 12 months after index angiography, this number was 92 254 (82.4%).

The number of patients prescribed any statin and high-intensity statin treatment regimen increased to 74 400 (68.7%) and 57 297 (52.9%), respectively, at 6 months and then declined to 42 281 (65.7%) and 33 048 (51.4%), respectively, at 3 years (eTable 2 in Supplement 1). Use of ezetimibe remained low throughout the observation period (1168 patients [1.1%] at 6 months and 1104 patients [1.7%] at 3 years). Any statin regimen and high-intensity statin use were higher among patients with ACS vs patients without ACS at most time points. Six months after baseline angiography, mean (SD) and median (IQR) LDL-C levels were 80.4 (42.8) mg/dL and 71.4 (52.0-97.6) mg/dL, respectively, and the number of patients with LDL-C levels of 70 mg/dL or more decreased to 56 405 patients (52.1%). The findings 3 years after baseline were similar (eTable 3 in Supplement 1).

At 12 months, LDL-C levels of more than 70 mg/dL were observed in a higher proportion of Black patients and female patients, whereas LDL-C levels of less than 55 mg/dL were observed in a higher proportion of White, Hispanic, and male patients (eTable 4 in Supplement 1). Patients with LDL-C levels of less than 55 mg/dL had more comorbidities and more frequently had obstructive CAD than patients with LDL-C levels of 55 mg/dL or more. However, the anatomic complexity of CAD, as calculated by the VA SYNTAX score,^19,20^ was similar among patients with LDL-C levels of less than 55 mg/dL, between 55 mg/dL and 70 mg/dL, and more than 70 mg/dL at 12 months.

During observation of up to 4 years, there were a total of 20 332 deaths, 4663 MIs, 1962 strokes, and 15 229 coronary revascularization procedures. Coronary revascularization was the most common event during years 1 and 2, while death was the most common event during years 3 and 4 (eTable 5 in Supplement 1).

### Optimized LLT, Projected LDL-C, and Projected Outcomes

**Figure 2.**
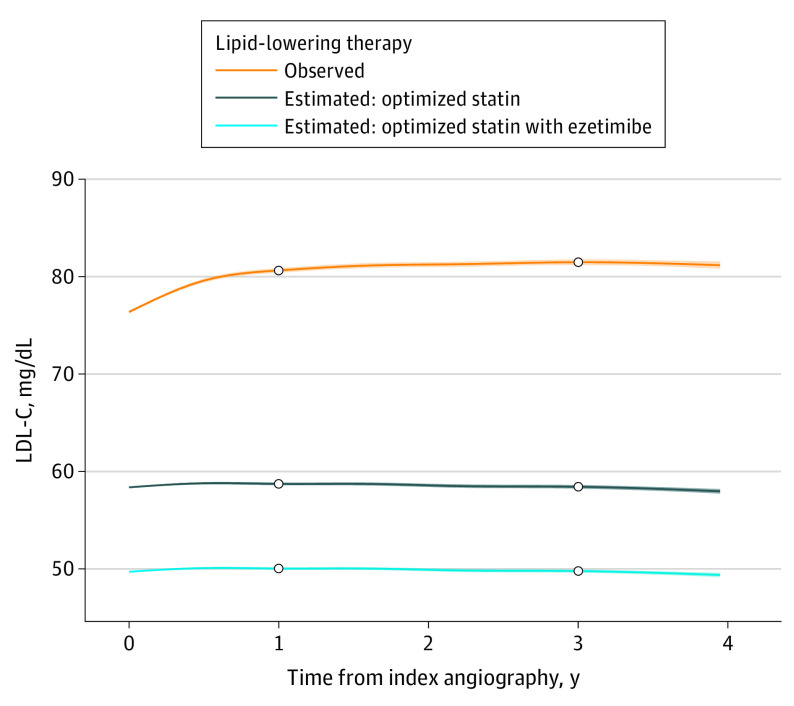
Observed and Projected Low-Density Lipoprotein Cholesterol (LDL-C) Trajectories The mean LDL-C levels and 95% CIs for observed lipid-lowering therapy are presented, along with those projected for an optimized statin regimen alone and for an optimized statin regimen with ezetimibe. Compared with observed LDL-C levels, optimized statin treatment was associated with a 21.9 mg/dL (to convert to millimoles per liter, multiply by 0.0259) projected reduction at 1 year and a 23.0 mg/dL projected reduction at 3 years. Compared with optimized statin therapy alone, the addition of ezetimibe was associated with an additional 8.7 mg/dL projected LDL-C value reduction at both 1 and 3 years, corresponding to 30.6 mg/dL and 31.7 mg/dL reductions from observed levels. Thus, the addition of ezetimibe to an optimized statin regimen resulted in a further 39.7% and 37.8% reduction of LDL-C levels of the reductions achieved by optimized statin alone at 1 and 3 years, respectively.

The projected effects of optimized statin therapy alone or with ezetimibe on LDL-C were modeled for each patient in the cohort. A total of 93 915 patients (83.9%) were deemed eligible for an 80-mg atorvastatin dosage daily, and 17 183 patients (15.3%) were deemed eligible for a 20-mg atorvastatin dosage daily. A total of 856 patients (0.76%) were deemed ineligible for statin treatment. The statistical models used measured LDL-C values for 67% and imputed LDL-C values for 33% of the study period. Figure 2 shows the differences in LDL-C levels over time between observed LLT and optimized statin therapy alone or with ezetimibe. Compared with observed LLT, optimized statin treatment alone was associated with a projected mean LDL-C reduction of 21.9 mg/dL at 1 year and a mean LDL-C reduction of 23.0 mg/dL at 3 years. Compared with optimized statin alone, addition of ezetimibe was associated with additional projected mean (SD) LDL-C reduction of 8.7 (0.012) mg/dL at 1 year and 8.7 (0.015) at 3 years, corresponding to total mean (SD) LDL-C reductions of 30.6 (0.083) mg/dL and 31.7 (0.098) mg/dL at 1 and 3 years. Thus, on a cohort level, adding ezetimibe to optimized statin was projected to provide an additional reduction of LDL-C concentration approximately 40% as great as the reduction from augmenting observed statin therapy to optimized statin therapy.

**Figure 3.**
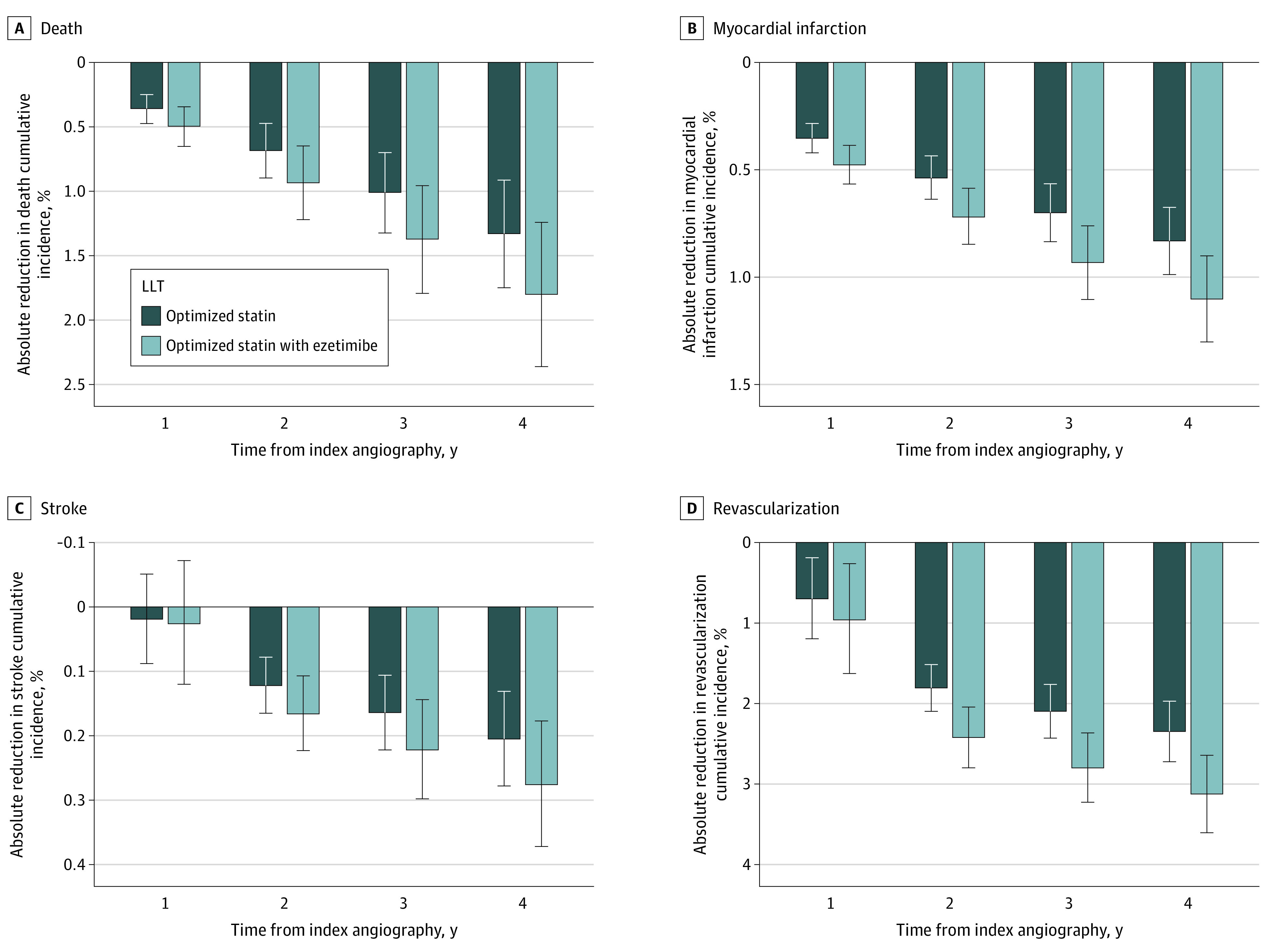
Projected Absolute Reduction in Cumulative Incidence of Adverse Outcomes with Optimized Lipid-Lowering Therapy (LLT) The projected percentage of risk reduction is presented for the cumulative incidences of the following 4 subcategories of adverse events: A, death; B, myocardial infarction; C, stroke; and D, coronary revascularization. The error bars represent 95% CIs. The values were projected for optimized statin therapy alone and for optimized statin therapy with ezetimibe.

Figure 3 shows the projected absolute reduction in the cumulative incidence of death and cardiovascular events with optimized statin therapy alone or with ezetimibe added, compared with observed LLT. At 1 year, the projected reduction in the incidence of death was 0.36% (95% CI, 0.25%-0.47%) with optimized statin therapy alone and 0.50% (95% CI, 0.34%-0.65%) with optimized statin therapy and ezetimibe (eTable 6 in Supplement 1). At 4 years, these reductions were 1.33% (95% CI, 0.91%-1.75%) and 1.80% (95% CI, 1.24%-2.36%), respectively. Commensurate incremental benefit of ezetimibe was projected for the risks of MI, stroke, and coronary revascularization (eFigure 2 in Supplement 1). The projected absolute reduction in the risk of death with optimized LLT at 4 years was greater among those without ACS vs those with ACS (eTable 7 in Supplement 1).

Table 2 indicates that, under the assumptions of the analysis, 1 death, MI, stroke, or coronary revascularization could be prevented in 4 years by augmenting statin therapy from observed to optimized levels in 76, 121, 488, and 43 patients, respectively. With the further addition of ezetimibe, the same benefits could be achieved through treatment of 56, 91, 363, and 33 patients, respectively.

**Table 2.** NNT to Prevent 1 Adverse Event by Optimizing Lipid-Lowering Therapy^a^

Time from index angiography	Death, NNT (95% CI)	Myocardial infarction, NNT (95% CI)	Stroke, NNT (95% CI)	Coronary revascularization, NNT (95% CI)
**Optimized statin therapy only**				
1 Year	280 (211-403)	284 (238-353)	NA	144 (84-532)
2 Years	147 (112-212)	186 (158-230)	818 (606-1279)	56 (48-66)
3 Years	100 (76-143)	143 (120-177)	610 (450-945)	48 (42-57)
4 Years	76 (58-110)	121 (102-149)	488 (360-767)	43 (37-51)
**Optimized statin therapy + ezetimibe**				
1 Year	203 (154-291)	210 (177-260)	NA	105 (62-383)
2 Years	108 (82-155)	139 (119-171)	603 (449-934)	42 (36-49)
3 Years	73 (56-105)	108 (91-132)	452 (336-695)	36 (31-43)
4 Years	56 (43-81)	91 (77-112)	363 (269-565)	33 (28-38)

Abbreviations: NA, not applicable because 95% CI spanned 0; NNT, number needed to treat.

^a^ Data are derived from linear mixed models using 1000 bootstrap samples as described in the Methods section.

## Discussion

In this cohort study among US military veterans with CAD, baseline LLT was often suboptimal. Six months after documentation of obstructive or nonobstructive CAD by coronary angiography, nearly one-third of patients remained untreated with a statin, and only slightly more than half received high-intensity statin treatment. Use of ezetimibe was extremely low at baseline and showed little growth over time. Robust modeling identified unrealized opportunities for substantial reductions in death and cardiovascular events through the use of optimized statin treatment, enhanced further with the use of ezetimibe.

Although the LLT used in clinical practice among this cohort of US veterans with CAD was suboptimal, it was superior to the LLT used in a large private-sector cohort of more than 100 000 patients with ASCVD (any statin, 53%; high-intensity statin regimen, 15%) and in a recent NHANES survey cohort of 1176 patients with established ASCVD (any statin, 58%).^13,21^ Medication copayments, known to be associated with the prescription of medications and patient adherence,^22,23,24,25^ may help explain greater use of evidence-based LLT in veterans. Recipients of VA health care have monthly copayments for atorvastatin and ezetimibe of $0 to $5 (depending on income and service-connected disability rating).^26^

This analysis found that patients with cardiovascular comorbidities and obstructive CAD were more likely to achieve guideline-recommended LDL-C goals. This suggests that clinicians may prioritize LLT in such patients and underappreciate the potential benefit of LLT in patients with nonobstructive CAD or fewer comorbid conditions.^27^ The analysis also identified disparities by race, ethnicity, and sex in achievement of LDL-C goals, with less frequent achievement in Black and female patients than in White, Hispanic, and male patients. This finding is congruent with other studies demonstrating lower rates of statin use among Black persons vs White persons, despite a higher burden of cardiovascular disease in some racial and ethnic minority communities.^28,29,30^ These findings call for targeted interventions to promote greater equity in the secondary prevention of cardiovascular disease.^31^

The present analysis identified substantial opportunity to intensify LLT and thus further reduce the risk of death and cardiovascular events in veterans with angiographically proven CAD. In absolute terms, intensification of LLT by optimizing statin therapy was most important, with the addition of ezetimibe projected to contribute notable incremental benefit.

In addition to underuse of statins, the adjunctive use of ezetimibe was low. Similar observations were reported from the GOULD registry and recent clinical trials of PCSK9 inhibitors where use of ezetimibe was less than 10%,^9,10,11,32^ despite data identifying ezetimibe as a cost-effective therapy.^33,34^ Underuse of ezetimibe may reflect its modest benefit when added to statin therapy on MI, stroke, and coronary revascularization and no reduction in death in the IMPROVE-IT trial.^5^ However a subsequent meta-analysis of LLT trials indicated a consistent reduction in the risk of major vascular events per 38.67 mg/dL reduction in LDL-C levels with statins (RR, 0.78; 95% CI, 0.65-0.94), ezetimibe (RR, 0.79; 95% CI, 0.67-0.93), and PCSK9 inhibitors (RR, 0.80; 95% CI, 0.61-1.04), suggesting that LDL-C reduction drives improved clinical outcomes irrespective of the mechanism of reduction.^6^ Thus, the low prevalent use of ezetimibe in the current analysis illustrates an opportunity to achieve meaningful incremental clinical benefit by prescribing a relatively weak lipid-lowering agent to a large segment of the population at risk but untreated. Indeed, projected reductions in the 4-year risk of cardiovascular events were approximately 35% greater with optimized statin plus ezetimibe, compared with optimized statin alone (Figure 3; eTable 6 in Supplement 1).

### Limitations

The findings of this study must be interpreted within its limitations. Potential reduction of death with ezetimibe is assumed from its reduction of LDL-C applied to the corresponding CTT formula; however, the only available randomized placebo-controlled trial to date has demonstrated reduction of death by ezetimibe. The CTT formulas were developed from clinical trials in highly selected populations with adjudicated outcomes, which included patients with or without established ASCVD.^4^ The accuracy of those formulae in the present analysis cohort is uncertain. It is likely that the veteran cohort had different competing risks for death than the trial populations used to derive the CTT formulas, as evidenced by the comparatively high rates of death compared with cardiovascular events apparent in the present results. The administrative data used to ascertain outcomes in the present analysis did not allow for reliable classification of causes of death (ie, cardiovascular). Further, ascertainment of events from administrative data elements may differ from ascertainment by adjudication in clinical trials. Despite these caveats, the CTT formulas have been applied successfully to other observational cohorts.^13^ Imperfect alignment of laboratory testing with changes in LLT necessitated imputation of some LDL-C values. The mean percentage reduction of LDL-C levels associated with a given dose of atorvastatin or ezetimibe was used to estimate the association of changes in LLT regimens with changes in LDL-C levels; thus, interindividual variability in response to LLT was not considered. Moreover, the analysis assumed that filled and released medications were taken as prescribed and did not consider the potential effects of suboptimal adherence. To the extent that some patients were comanaged by other health care systems, some laboratory, pharmacy, and outcomes data may not have been available, and the potential effects of medications prescribed by non-VA clinicians were not considered. The analysis cohort consisted only of patients who had CAD identified by direct coronary angiography, and thus underestimated the total number of veterans with CAD or other ASCVD who might benefit from intensified LLT. In addition, there was a paucity of women in the analysis cohort, which is consistent with prior studies of the VA population. Furthermore, racial and ethnic minority populations in this VA health care system cohort were not representative of the total US population.

## Conclusions

In this cohort study, insufficient LLT was common among veterans with CAD. These patients could potentially reap sizeable reductions in the risks of death and cardiovascular events through widespread use of optimized statin therapy. Ezetimibe is associated with lesser reductions in LDL-C levels than statins, but its use among veterans was much lower than that of statins. Therefore, on a population level, broad adjunctive use of ezetimibe may provide meaningful incremental clinical benefit compared with optimized statin therapy alone.

## Notes

Supplement 1. **eMethods.** Supplemental Methods **eTable 1.** Relationship Between Prescribed Statin Intensity and Anticipated LDL-C Reduction **eTable 2.** Lipid-Lowering Therapies at Baseline and Following Index Angiography for the Overall, Non-ACS, and ACS Cohorts **eTable 3.** LDL-C with Observed Lipid-Lowering Therapies at Baseline, 6 Months, and 3 Years After Index Angiography **eTable 4.** Baseline Characteristics Stratified by Achievement of LDL-C <55 mg/dL, 55-70 mg/dL, or >70 mg/dL at 12 Months **eTable 5.** Observed Cumulative Incidence and Cumulative Number of Events **eTable 6.** Projected Reductions in Cumulative Incidence of Adverse Events with Intensified Lipid-Lowering Therapy **eTable 7.** Projected Reductions in Cumulative Incidence of Events with Optimized Statin and Ezetimibe by Non-ACS or ACS Clinical Presentation **eFigure 1.** Flow Diagram for the Formation of the Analytic Cohort **eFigure 2.** Estimated Cumulative Incidence of Adverse Outcomes with Observed and Optimized Lipid-Lowering Therapy

Supplement 2.
Data Sharing Statement
