# Supplementary material for: Projected Outcomes of Optimized Statin and Ezetimibe Therapy in US Military Veterans with Coronary Artery Disease
Source: JAMA Netw Open. 2023 Aug 28;6(8):e2329066. doi: 10.1001/jamanetworkopen.2023.29066 (PMC10463102; doi:10.1001/jamanetworkopen.2023.29066)
Supplement: Supplement 1. — eMethods. Supplemental Methods eTable 1. Relationship Between Prescribed Statin Intensity and Anticipated LDL-C Reduction eTable 2. Lipid-Lowering Therapies at Baseline and Following Index Angiography for the Overall, Non-ACS, and ACS Cohorts eTable 3. LDL-C with Observed Lipid-Lowering Therapies at Baseline, 6 Months, and 3 Years After Index Angiography eTable 4. Baseline Characteristics Stratified by Achievement of LDL-C <55 mg/dL, 55-70 mg/dL, or >70 mg/dL at 12 Months eTable 5. Observed Cumulative Incidence and Cumulative Number of Events eTable 6. Projected Reductions in Cumulative Incidence of Adverse Events with Intensified Lipid-Lowering Therapy eTable 7. Projected Reductions in Cumulative Incidence of Events with Optimized Statin and Ezetimibe by Non-ACS or ACS Clinical Presentation eFigure 1. Flow Diagram for the Formation of the Analytic Cohort eFigure 2. Estimated Cumulative Incidence of Adverse Outcomes with Observed and Optimized Lipid-Lowering Therapy [file jamanetwopen-e2329066-s001.pdf]

Supplemental Online Content

Kovach CP, Mesenbring EC, Gupta P, et al. Cardiovascular risk assessment with optimized statin and ezetimibe therapy in US military veterans with coronary artery disease. *JAMA Netw Open.* 2023;6(8):e2329066.
doi:10.1001/jamanetworkopen.2023.29066

eMethods. Supplemental Methods

eTable 1. Relationship Between Prescribed Statin Intensity and Anticipated LDL-C Reduction

eTable 2. Lipid-Lowering Therapies at Baseline and Following Index Angiography for the Overall, Non-ACS, and ACS Cohorts

eTable 3. LDL-C with Observed Lipid-Lowering Therapies at Baseline, 6 Months, and 3 Years After Index Angiography

eTable 4. Baseline Characteristics Stratified by Achievement of LDL-C <55 mg/dL, 55-70 mg/dL, or >70 mg/dL at 12 Months

eTable 5. Observed Cumulative Incidence and Cumulative Number of Events

eTable 6. Projected Reductions in Cumulative Incidence of Adverse Events with Intensified Lipid-Lowering Therapy

eTable 7. Projected Reductions in Cumulative Incidence of Events with Optimized Statin and Ezetimibe by Non-ACS or ACS Clinical Presentation

eFigure 1. Flow Diagram for the Formation of the Analytic Cohort

eFigure 2. Estimated Cumulative Incidence of Adverse Outcomes with Observed and Optimized Lipid-Lowering Therapy

This supplemental material has been provided by the authors to give readers additional information about their work.

© 2023 Kovach CP et al. *JAMA Network Open.*

SUPPLEMENTAL METHODS

Inclusion and Exclusion Criteria

Included patients ≥18 years of age who underwent coronary angiography demonstrating obstructive or non-obstructive CAD during the study period. Obstructive CAD was defined as ≥50% stenosis of the left main or ≥70% stenosis of the left anterior descending, left circumflex, or right coronary arteries. Non-obstructive CAD was defined as ≥20% stenosis in a coronary artery that did not meet the definition of obstructive CAD. The severity of CAD was further described using the VA SYNTAX score, an automatically calculated anatomic disease severity score [19,20]. Included patients had an LDL-C measurement within 12 months preceding index angiography or an LDL-C measurement within 3 months following index angiography if they were statin naïve at time of index angiography. Each LDL-C measurement was aligned with concurrent LLT using the VA pharmacy database. Patients were excluded if they had no coronary stenosis ≥20%, insufficient laboratory or pharmacy data for analysis, were treated with PCSK9 inhibitors, had eGFR <15 mL/min or dialysis-dependent chronic kidney disease at the time of index angiography, or died within 30-days of index angiography. Patients who were prescribed a non-standard statin dose (i.e., a dose and/or frequency not described in Supplemental Table 1) were also excluded.

Study Design

The analysis proceeded in four steps. First, baseline LDL-C and LLT (at the time of index angiography) was defined. Second, observed LDL-C, LLT, and clinical outcomes were determined throughout the subsequent observation period for each patient and compiled for the overall cohort. Third, time-varying LDL-C was projected for each patient and for the full cohort based on expected lipid-lowering effects of optimized statin therapy, either alone or in conjunction with ezetimibe. Fourth, the CTT formulae relating reduction in LDL-C to reduction in the risk of

© 2023 Kovach CP et al. *JAMA Network Open.*

adverse events were applied to calculate potential reductions in clinical outcomes achievable with optimized statin therapy alone or with the addition of ezetimibe [4].

Projected LDL-C with Optimized Lipid-Lowering Therapy

For a patient without contraindications, optimized statin was considered high-intensity treatment with atorvastatin 80mg daily and assumed to result in a 54% LDL-C reduction from an untreated baseline. For patients >80 years of age, with body mass index <20 kg/m2, chronic HIV infection, or treated with amiodarone, diltiazem, verapamil, or cyclosporine, optimized statin was considered moderate-intensity treatment with atorvastatin 20mg and assumed to result in a 41% LDL-C reduction from an untreated baseline. For patients with documented statin intolerance (defined as ≥2 statins listed as producing allergies or adverse reactions), no statin treatment was considered optimized. Ezetimibe 10mg was considered an appropriate addition to therapy in all cases where it was not already prescribed, and was assumed to result in a further 15% reduction in LDL-C from the level on optimized statin[8].

For each patient, the time-varying LDL-C levels that could have been achieved with optimized statin and ezetimibe treatment were estimated. First, a standard nomogram relating statin agent and dose to mean LDL-C reduction from an untreated baseline (Supplemental Table 1) was applied to 'reverse estimate' the untreated baseline LDL-C based on the patients' active LLT. For example, consider a patient with a laboratory LDL-C measurement of 100 mg/dl on treatment with atorvastatin 20mg daily. Atorvastatin 20mg is assumed to have reduced LDL-C by 41% from an untreated baseline (Supplemental Table 1). Thus, the patient's untreated baseline LDL-C is 100 ÷ 0.59 = 169.5 mg/dl. Next, the impact of optimized LLT on LDL-C levels was estimated. Optimal LLT was considered high-intensity statin (atorvastatin 80mg) and ezetimibe (10mg) for all patients without known contraindications. The example patient has no contraindication to high-intensity statin. Atorvastatin 80mg is assumed to produce a 54%

© 2023 Kovach CP et al. *JAMA Network Open.*

reduction in LDL-C from an untreated baseline (Supplemental Table 1)[8]. Therefore, the substitution of atorvastatin 80 mg for atorvastatin 20 mg is projected to lower the untreated baseline LDL-C from 169.5 mg/dl to 78 mg/dl (i.e., 169.5 mg/dl x 0.46 = 77.7 mg/dl). Ezetimibe was assumed to produce a 15% reduction of LDL-C in addition to intensive statin treatment [5]. For the example patient, a 15% further reduction to 66 mg/dl is projected (i.e., 77.7 mg/dl x 0.85 = 66.0 mg/dl). In this manner, a 34 mg/dL (0.88 mmol/L) reduction in LDL-C with optimal therapy (atorvastatin 80mg and ezetimibe 10mg), as compared to baseline therapy (atorvastatin 20mg), is estimated. For each patient in the analysis, this procedure was repeated at each time point using the reductions in LDL-C achieved with high-intensity statin and ezetimibe as compared to their observed LLT regimen. If a patient had a contraindication to high-intensity statin treatment, then reductions in LDL-C achieved with moderate-intensity statin (i.e., atorvastatin 20 mg) and ezetimibe were assumed. If a patient had a contraindication to any statin treatment, only the LDL-C reduction associated with ezetimibe was assumed. Using the same methods, aside from ezetimibe calculations, time-varying LDL-C levels that could have been achieved with optimized statin only treatment were additionally estimated.

Outcomes

Coronary revascularization was defined as PCI or CABG occurring >30 days after index angiography to account for potential staged or urgent procedures [4]. Death was ascertained from VA administrative records. Other outcomes were ascertained through a review of administrative diagnosis and procedure codes within the VA Healthcare System and the VA Community Care Network.

Statistical Analysis

© 2023 Kovach CP et al. *JAMA Network Open.*

The potential impact of optimized LLT with statin and with statin plus ezetimibe on outcomes was estimated by applying the relevant CTT formulas for rate ratio reduction in death and other outcomes to the observed cumulative incidences of death and other outcomes in each 6-month time interval of the analysis [4]. Modelled rate ratios (RR) for outcomes were calculated as: $(RR_{CTT})^{\text{net LDL-C lowering in mmol/L}} = RR_{model}$ using simulated rate ratios for RR_{CTT} mimicking those for all-cause death, non-fatal MI, stroke, and revascularization observed in the CTT meta-analysis[4] described below:

1. All-cause death, RR 0.90, 95% CI [0.87-0.93], per 1 mmol/L reduction in LDL-C
2. Non-fatal MI, RR 0.74, 95% CI [0.69-0.78], per 1 mmol/L reduction in LDL-C
3. Coronary revascularization (PCI or CABG)
    a. Overall, RR 0.76, 95% CI [0.73-0.80], per 1 mmol/L reduction in LDL-C
    b. Year 0-1, RR 0.88, 95% CI [0.80-0.97], per 1 mmol/L reduction in LDL-C
    c. Year 1-2, RR 0.75, 95% CI [0.67-0.84], per 1 mmol/L reduction in LDL-C
    d. Year 2-3, RR 0.72, 95% CI [0.64-0.81], per 1 mmol/L reduction in LDL-C
    e. Year 3-4, RR 0.64, 95% CI [0.56-0.73], per 1 mmol/L reduction in LDL-C
    f. Year 4-5, RR 0.70, 95% CI [0.61-0.80], per 1 mmol/L reduction in LDL-C
    g. Year 5+, RR 0.73, 95% CI [0.60-0.89], per 1 mmol/L reduction in LDL-C
4. Stroke
    a. Overall, RR 0.85, 95% CI [0.80-0.90], per 1 mmol/L reduction in LDL-C.
    b. Year 0-1, RR 0.96, 95% CI [0.82-1.12], per 1 mmol/L reduction in LDL-C
    c. Year 1-2, RR 0.77, 95% CI [0.66-0.91], per 1 mmol/L reduction in LDL-C
    d. Year 2-3, RR 0.83, 95% CI [0.70-0.98], per 1 mmol/L reduction in LDL-C
    e. Year 3-4, RR 0.79, 95% CI [0.66-0.95], per 1 mmol/L reduction in LDL-C
    f. Year 4-5, RR 0.87, 95% CI [0.70-1.06], per 1 mmol/L reduction in LDL-C
    g. Year 5+, RR 0.82, 95% CI [0.61-1.11], per 1 mmol/L reduction in LDL-C

***A 1 mmol/L reduction in LDL-C is equivalent to 38.67 mg/dL.

For example, assume that the actual observed LDL-C is 100 mg/dL at the first half of year 2 of the observation period and projected optimized LDL-C is 66 mg/dL, for a net reduction of 34 mg/dL (0.88 mmol/L) with optimized lipid-lowering therapy. The observed mortality cumulative incidence

© 2023 Kovach CP et al. *JAMA Network Open.*

at 2.5 years is 1%. The CTT formula predicts that a 0.88 mmol/L reduction in LDL-C would result in a rate ratio of mortality of $0.90^{0.88}$ = 0.91. Thus, the projected incidence of mortality with optimized LLT would be 1.0 x 0.91 = 0.91%. Using this method for each time interval, projected cumulative incidence of death and rehospitalization for MI were plotted at 6-month intervals. The formula below was used to calculate projected cumulative event incidence using the expected reduction in LDL-C with augmentation of LLT and the observed cumulative incidence for the outcome *i* at time point *j* in bootstrap model *k*. Distributions of the values used for $RR_{i,j,k}$ (rate ratio per 1 mmol/L reduction in LDL-C) were within 0.15% of those reported in the CTT analysis.

$$Projected\ Cumulative\ Incidence_{i,j,k} = Observed\ Cumulative\ Incidence_{i,j,k} * (RR_{i,j,k})^{LDL-C\ reduction\ in\ mmol/L_{j,k}}$$

For outcomes with treatment year-specific rate ratios (e.g., coronary revascularization, stroke), the year 0-1 RR was used to model RRs in the first year following index angiography and the overall RR was used for all subsequent treatment years (i.e., ≥1 year). For example, assume a net 34 mg/dL (0.88 mmol/L) reduction with optimized LLT at year 1 and the net reduction is still 34 mg/dL at year 2. Modelled RR for coronary revascularization was estimated as $0.88^{0.88}$=0.89 at year 1 and $0.76^{0.88}$=0.79 at year 2. If the observed cumulative incidence of coronary revascularization in this example was 3% at year 1 and 5% at year 2, then the projected cumulative incidence of coronary revascularization with optimized LLT would be 3.0 x 0.89 = 2.67% at year 1 and 5.0 x 0.79 = 3.95% at year 2. Using this method for each time interval, projected cumulative incidence curves for outcomes with treatment year-specific risk-ratios (e.g., coronary revascularization, stroke) were constructed at 6-month intervals.

© 2023 Kovach CP et al. *JAMA Network Open.*

SUPPLEMENTAL TABLES

Supplemental Table 1. Relationship between prescribed statin intensity and anticipated LDL-C reduction.

% LDL Reduction	Ezetimibe	Simvastatin	Atorvastatin	Lovastatin	Pravastatin	Fluvastatin	Rosuvastatin	Pitavastatin
15	10 mg							
27		10mg‡	-	20mg‡	20mg‡	40mg‡	-	-
34		20mg†	10mg†	40mg†	40mg†	80mg†	-	1mg‡
41		40mg†	20mg†	80mg†	80mg†	-	-	2mg†
48		80mg*	40mg*	-	-	-	10mg†	4mg†
54		-	80mg*	-	-	-	20mg*	-
60		-	-	-	-	-	40mg*	-

Above table is adapted from: https://www.ncbi.nlm.nih.gov/books/NBK395573/ [8]. High, medium, and low intensity statin indicated with *, †, and ‡, respectively.

© 2023 Kovach CP et al. *JAMA Network Open.*

Supplemental Table 2. Lipid-Lowering Therapies at Baseline and following Index Angiography for the Overall, Non-ACS, and ACS Cohorts.

**Overall Cohort**	**Baseline (N=111,954)**	**3 months (N=111,954)**	**6 months (N=108,320)**	**1 year (N=105,082)**	**2 years (N=86,864)**	**3 years (N=64,326)**	**4 years (N=43,794)**
Statin							
Overall	66,877 (59.7)	82749 (75.0)	74,400 (68.7)	70,969 (67.5)	57,710 (66.4)	42,281 (65.7)	28,634 (65.4)
Low Intensity	2,886 (2.6)	1589 (1.4)	1,401 (1.3)	1,211 (1.2)	939 (1.1)	631 (1.0)	380 (0.9)
Moderate Intensity	24,949 (22.3)	17354 (15.7)	15,702 (14.5)	14,494 (13.8)	11,705 (13.5)	8,602 (13.4)	5,751 (13.1)
High Intensity	39,042 (34.9)	63806 (57.8)	57,297 (52.9)	55,264 (52.6)	45,066 (51.9)	33,048 (51.4)	22,503 (51.4)
Ezetimibe	623 (0.6)	1135 (1.0)	1,168 (1.1)	1,417 (1.3)	1,324 (1.5)	1,104 (1.7)	909 (2.1)
**Non-ACS Cohort**	**Baseline (N=81,917)**	**3 months (N=81,917)**	**6 months (N=79,357)**	**1 year (N=77,069)**	**2 years (N=63,617)**	**3 years (N=46,439)**	**4 years (N=31,103)**
Statin							
Overall	51,259 (62.6)	59146 (73.2)	54,071 (68.1)	51,995 (67.5)	42,349 (66.6)	30,649 (66.0)	20,332 (65.4)
Low Intensity	2,212 (2.7)	1369 (1.7)	1,223 (1.5)	1,039 (1.3)	818 (1.3)	553 (1.2)	329 (1.1)
Moderate Intensity	19,181 (23.4)	14513 (18.0)	13,165 (16.6)	12,202 (15.8)	9,686 (15.2)	7,026 (15.1)	4,609 (14.8)
High Intensity	29,866 (36.5)	43264 (53.6)	39,683 (50.0)	38,754 (50.3)	31,845 (50.1)	23,070 (49.7)	15,394 (49.5)
Ezetimibe	479 (0.6)	823 (1.0)	847 (1.1)	1,017 (1.3)	947 (1.5)	772 (1.7)	617 (2.0)
**ACS Cohort**	**Baseline (N=30,037)**	**3 months (N=30,037)**	**6 months (N=28,963)**	**1 year (N=28,013)**	**2 years (N=23,247)**	**3 years (N=17,887)**	**4 years (N=12,691)**
Statin							
Overall	15,618 (52.0)	23603 (79.9)	20,329 (70.2)	18,974 (67.7)	15,361 (66.1)	11,632 (65.0)	8,302 (65.4)
Low Intensity	674 (2.2)	220 (0.7)	178 (0.6)	172 (0.6)	121 (0.5)	78 (0.4)	51 (0.4)
Moderate Intensity	5,768 (19.2)	2841 (9.6)	2,537 (8.8)	2,292 (8.2)	2,019 (8.7)	1,576 (8.8)	1,142 (9.0)
High Intensity	9,176 (30.5)	20542 (69.6)	17,614 (60.8)	16,510 (58.9)	13,221 (56.9)	9,978 (55.8)	7,109 (56.0)
Ezetimibe	144 (0.5)	312 (1.1)	321 (1.1)	400 (1.4)	377 (1.6)	332 (1.9)	292 (2.3)

Data presented as N (%).

© 2023 Kovach CP et al. *JAMA Network Open.*

Supplemental Table 3. LDL-C with Observed Lipid-Lowering Therapies at Baseline, 6 Months, and 3 Years after Index Angiography.

**Baseline**	**Overall (N=111,954)**	**Non-ACS (N=81,917)**	**ACS (N=30,037)**	**P-Value**
Overall, Median [IQR]	74.00 [55.12, 96.78]	74.00 [55.17, 97.00]	73.72 [55.00, 96.32]	0.174
Overall, Mean (SD)	79.70 (36.37)	79.82 (36.48)	79.40 (36.01)	0.086
Statin				
Low Intensity	72.01 [56.21, 90.80]	73.07 [57.00, 91.98]	69.14 [54.76, 86.11]	0.001
Moderate Intensity	69.60 [53.93, 87.00]	70.00 [54.12, 87.78]	68.00 [52.54, 85.38]	<0.001
High Intensity	66.04 [49.00, 86.00]	66.00 [49.00, 85.63]	66.79 [49.00, 87.00]	0.077
None	85.47 [64.00, 111.00]	87.00 [65.31, 113.00]	82.47 [61.60, 106.78]	<0.001
<70 mg/dL	49,743 (44.4)	36,252 (44.3)	13,491 (44.9)	0.050
**6 months after Index Angiography**	**Overall (N=108,320)**	**Non-ACS (N=79.357)**	**ACS (N=28,963)**	**P-Value**
Overall, Median [IQR]	71.40 [52.00, 97.63]	72.03 [53.00, 98.00]	69.00 50.00, 96.75]	<0.001
Overall, Mean (SD)	80.39 (42.78)	80.74 (42.01)	79.45 (44.81)	<0.001
Statin				
Low Intensity	77.00 [61.00, 95.88]	77.00 [61.21, 95.00]	77.59 [58.11, 97.71]	0.994
Moderate Intensity	68.72 [52.51, 87.00]	69.00 [53.00, 87.00]	67.05 [50.74, 86.73]	0.024
High Intensity	62.92 [46.22, 82.00]	63.37 [47.00, 82.00]	61.06 [45.00, 81.00]	<0.001
None	96.21 [69.94, 130.00]	96.15 [70.10, 128.85]	96.45 [68.20, 133.34]	0.652
Change in LDL-C from Baseline	-0.58 (39.93)	-0.85 (39.26)	0.15 (41.69)	<0.001
<70 mg/dL	51,915 (47.9)	37,211 (46.9)	14,704 (50.8)	<0.001
**3 years after Index Angiography**	**Overall (N=64,326)**	**Non-ACS (N=46,439)**	**ACS (N=17,887)**	**P-Value**
Overall, Median [IQR]	72.00 [52.00, 99.74]	72.60 [52.87, 99.20]	70.64 [51.00, 100.20]	<0.001
Overall, Mean (SD)	81.95 (45.64)	81.76 (44.31)	82.43 (48.93)	0.091
Statin				
Low Intensity	75.10 [58.00, 92.00]	75.16 [58.00, 92.00]	71.95 [56.42, 94.94]	0.897

© 2023 Kovach CP et al. *JAMA Network Open.*

Moderate Intensity	66.96 [50.83, 83.40]	67.60 [51.27, 84.00]	64.00 [48.10, 81.20]	<0.001
High Intensity	61.00 [45.00, 79.00]	61.72 [45.00, 79.00]	60.00 [44.00, 78.00]	<0.001
None	103.82 [76.00, 138.38]	103.00 [76.09, 137.00]	105.61 [75.00, 141.30]	0.012
Change in LDL-C from Baseline	-1.31 (48.52)	-0.79 (47.65)	-2.66 (50.67)	<0.001
<70 mg/dL	30,774 (47.3)	21,913 (46.6)	8,861 (49.0)	<0.001

Data are N (%) or median (IQR). P-values reflect comparisons between non-ACS and ACS patient categories.

© 2023 Kovach CP et al. *JAMA Network Open.*

Supplemental Table 4. Baseline Characteristics Stratified by Achievement of LDL-C <55 mg/dL, 55-70 mg/dL, or >70 mg/dL at 12 months.

	Overall (N = 105,082)	<55 mg/dL (N = 29,693)	55-70 mg/dL (N = 20,382)	>70 mg/dL (N = 55,007)	P-Value
**Demographics**					
Age	68.12 (8.71)	69.09 (8.31)	68.75 (8.45)	67.37 (8.94)	<0.001
Male	102,620 (97.7)	29,225 (98.4)	20,016 (98.2)	53,379 (97.0)	<0.001
Race					<0.001
White	85,918 (81.8)	24,719 (83.2)	17,026 (83.5)	44,173 (80.3)	
Black	16,537 (15.7)	4,173 (14.1)	2,856 (14.0)	9,508 (17.3)	
Other	2,627 (2.5)	801 (2.7)	500 (2.5)	1,326 (2.4)	
Hispanic	5,631 (5.4)	1,774 (6.0)	1,073 (5.3)	2,784 (5.1)	<0.001
**Medical History**					
CAD at index angiography					
Obstructive	71,019 (67.6)	21,368 (72.0)	14,232 (69.8)	35,419 (64.4)	<0.001
Non-obstructive	34,063 (32.4)	8,325 (28.0)	6,150 (30.2)	19,588 (35.6)	<0.001
VA SYNTAX	7 (2-17)	8 (2-18)	7 (1-17)	7 (1-16)	<0.001
Prior MI/PCI/CABG	50,856 (48.4)	15,139 (51.0)	10,175 (49.9)	25,542 (46.4)	<0.001
Prior MI	35,824 (34.1)	10,733 (36.1)	7,089 (34.8)	18,002 (32.7)	<0.001
Prior PCI	33,901 (32.3)	10,150 (34.2)	6,822 (33.5)	16,929 (30.8)	<0.001
Prior CABG	21,735 (20.7)	6,460 (21.8)	4,419 (21.7)	10,856 (19.7)	<0.001
Heart failure	31,546 (30.0)	9,666 (32.6)	6,064 (29.8)	15,816 (28.8)	<0.001
Prior CVA	10,186 (9.7)	3,115 (10.5)	1,955 (9.6)	5,116 (9.3)	<0.001
Peripheral artery disease	22,046 (21.0)	6,645 (22.4)	4,365 (21.4)	11,036 (20.1)	<0.001
Diabetes	53,478 (50.9)	17,368 (58.5)	10,678 (52.4)	25,432 (46.2)	<0.001
Chronic kidney disease	23,189 (22.1)	7,192 (24.2)	4,528 (22.2)	11,469 (20.9)	<0.001
Hypertension	95,774 (91.1)	27,604 (93.0)	18,715 (91.8)	49,455 (89.9)	<0.001
Hyperlipidemia	94,492 (89.9)	26,468 (89.1)	18,499 (90.8)	49,525 (90.0)	<0.001
Atrial fibrillation	18,875 (18.0)	5,889 (19.8)	3,727 (18.3)	9,259 (16.8)	<0.001
COPD	27,374 (26.1)	7,944 (26.8)	5,316 (26.1)	14,114 (25.7)	0.002
Obesity	52,571 (50.0)	15,147 (51.0)	10,307 (50.6)	27,117 (49.3)	<0.001
Sleep apnea	35,770 (34.0)	10,675 (36.0)	7,049 (34.6)	18,046 (32.8)	<0.001
Tobacco use	69,905 (66.5)	19,895 (67.0)	13,527 (66.4)	36,483 (66.3)	0.119
Alcohol abuse	9,934 (9.5)	2,661 (9.0)	1,730 (8.5)	5,543 (10.1)	<0.001
Other substance abuse	5,718 (5.4)	1,474 (5.0)	918 (4.5)	3,326 (6.0)	<0.001
Chronic HIV	668 (0.6)	161 (0.5)	126 (0.6)	381 (0.7)	0.03
**Selected Cardiovascular Medications**					
P2Y12 inhibitor	18,906 (18.0)	5,759 (19.4)	3,769 (18.5)	9,378 (17.0)	<0.001
Beta blocker	61,356 (58.4)	18,114 (61.0)	12,217 (59.9)	31,025 (56.4)	<0.001
CCB	27,687 (26.3)	8,097 (27.3)	5,484 (26.9)	14,106 (25.6)	<0.001
ACE/ARB/ARNI	56,677 (53.9)	17,036 (57.4)	11,370 (55.8)	28,271 (51.4)	<0.001

© 2023 Kovach CP et al. *JAMA Network Open.*

**Indication for Index Coronary Angiography**					<0.001
ACS					
Unstable angina	12,177 (11.6)	3,630 (12.2)	2,385 (11.7)	6,162 (11.2)	
NSTEMI	12,520 (11.9)	3,974 (13.4)	2,387 (11.7)	6,159 (11.2)	
STEMI	1,597 (1.5)	499 (1.7)	318 (1.6)	780 (1.4)	
Unspecified	962 (0.9)	309 (1.0)	185 (0.9)	468 (0.9)	
CCS					
Stable angina	23,266 (22.1)	6,406 (21.6)	4,673 (22.9)	12,187 (22.2)	
Atypical chest pain	12,205 (11.6)	3,212 (10.8)	2,283 (11.2)	6,710 (12.2)	
Unspecified	5,434 (5.2)	1,577 (5.3)	1,118 (5.5)	2,739 (5.0)	
Heart failure	2,022 (1.9)	639 (2.2)	381 (1.9)	1,002 (1.8)	
Cardiomyopathy	2,758 (2.6)	824 (2.8)	508 (2.5)	1,426 (2.6)	
Valve Disease	7,341 (7.0)	1,843 (6.2)	1,407 (6.9)	4,091 (7.4)	
Other	16,001 (15.2)	4,344 (14.6)	3,094 (15.2)	8,563 (15.6)	
Missing	8,799 (8.4)	2,436 (8.2)	1,643 (8.1)	4,720 (8.6)	

Abbreviations: ACEi, angiotensin-converting enzyme inhibitor; ARB, aldosterone receptor blocker; ARNI, angiotensin receptor-neprilysin inhibitor; CAD, coronary artery disease; COPD, chronic obstructive pulmonary disease; HIV, human immunodeficiency virus; MI, myocardial infarction; NSTEMI, non-ST-elevation MI; PCI, percutaneous coronary intervention; STEMI, ST-elevation MI; VA SYNTAX; Veterans Affairs Synergy between PCI with Taxus and Cardiac Surgery. Obesity was defined by a body mass index ≥30 kg/m^2. Tobacco use includes prior or current. Other indications for coronary angiography included arrhythmia, asymptomatic ischemic, cardiogenic shock, tamponade, congenital heart disease, pre-operative evaluation, pulmonary hypertension, syncope, transplant evaluation, and history of heart transplant. Data are N (%) or median (IQR) unless otherwise specified. P-values reflect comparisons among achieved LDL-C categories.

© 2023 Kovach CP et al. *JAMA Network Open.*

Supplemental Table 5. Observed Cumulative Incidence and Cumulative Number of Events.

Time from Angiography	Death		Myocardial Infarction		Stroke		Coronary Revascularization	
	Cumulative Incidence	Cumulative Events	Cumulative Incidence	Cumulative Events	Cumulative Incidence	Cumulative Events	Cumulative Incidence	Cumulative Events
1 Year	6.15 [6.01, 6.29]	6,887	2.25 [2.16, 2.33]	2,460	0.80 [0.75, 0.85]	875	9.91 [9.73, 10.09]	10,920
2 Years	11.36 [11.18, 11.55]	12,387	3.32 [3.21, 3.43]	3,499	1.34 [1.27, 1.41]	1,396	12.12 [11.92, 12.31]	13,058
3 Years	16.49 [16.26, 16.72]	16,894	4.25 [4.12, 4.38]	4,211	1.77 [1.68, 1.85]	1,725	13.86 [13.65, 14.07]	14,374
4 Years	21.58 [21.30, 21.85]	20,322	5.01 [4.87, 5.16]	4,633	2.20 [2.10, 2.29]	1,962	15.43 [15.19, 15.66]	15,229

Cumulative incidence data are percent [95% CI]. Cumulative numbers of observed events were ascertained from the analytic cohort of 111,954 patients.

© 2023 Kovach CP et al. *JAMA Network Open.*

Supplemental Table 6. Projected Reductions in Cumulative Incidence of Adverse Events with Intensified Lipid-Lowering Therapy

Time from Index Angiography	Death	Myocardial Infarction	Stroke	Coronary Revascularization
Optimized Statin Only				
1 Year	0.358 [0.249, 0.474]	0.353 [0.284, 0.421]	0.019 [-0.051, 0.088]	0.70 [0.19, 1.20]
2 Years	0.684 [0.473, 0.897]	0.538 [0.435, 0.637]	0.122 [0.078, 0.165]	1.81 [1.52, 2.10]
3 Years	1.009 [0.700, 1.324]	0.700 [0.568, 0.835]	0.164 [0.106, 0.222]	2.10 [1.76, 2.43]
4 Years	1.329 [0.913, 1.749]	0.831 [0.675, 0.988]	0.205 [0.131, 0.278]	2.35 [1.97, 2.72]
Optimized Statin + Ezetimibe				
1 Year	0.50 [0.34, 0.65]	0.48 [0.39, 0.57]	0.03 [-0.07, 0.12]	0.96 [0.26, 1.63]
2 Years	0.93 [0.65, 1.22]	0.72 [0.59, 0.85]	0.17 [0.11, 0.22]	2.42 [2.05, 2.80]
3 Years	1.37 [0.96, 1.79]	0.93 [0.76, 1.10]	0.22 [0.14, 0.30]	2.80 [2.37, 3.23]
4 Years	1.80 [1.24, 2.36]	1.10 [0.90, 1.30]	0.28 [0.18, 0.37]	3.12 [2.64, 3.61]

Data are percent [95% CI] absolute reduction in the cumulative incidence of events, derived from linear mixed models employing 1000 bootstrap samples as described in Methods.

© 2023 Kovach CP et al. *JAMA Network Open.*

Supplemental Table 7. Projected Reductions in Cumulative Incidence of Events with Optimized Statin and Ezetimibe by Non-ACS or ACS Clinical Presentation.

	Time	Death	Myocardial Infarction	Stroke	Coronary Revascularization
Non-ACS	1 Year	0.464 [0.322, 0.612]	0.304 [0.250, 0.361]	0.023 [0.064, 0.106]	0.969 [0.261, 1.669]
	2 Years	0.849 [0.589, 1.119]	0.457 [0.375, 0.540]	0.140 [0.093, 0.191]	2.270 [1.918, 2.625]
	3 Years	1.069 [0.738, 1.414]	0.527 [0.429, 0.624]	0.156 [0.103, 0.212]	2.220 [1.867, 2.565]
	4 Years	1.416 [0.978, 1.871]	0.641 [0.521, 0.755]	0.196 [0.130, 0.268]	2.471 [2.082, 2.877]
ACS	1 Year	0.503 [0.344, 0.662]	0.866 [0.703, 1.027]	0.030 [0.086, 0.143]	0.801 [0.217, 1.371]
	2 Years	0.893 [0.621, 1.169]	1.224 [0.993, 1.45]	0.190 [0.121, 0.260]	2.175 [1.824, 2.519]
	3 Years	0.849 [0.585, 1.118]	1.033 [0.841, 1.231]	0.175 [0.110, 0.243]	1.767 [1.482, 2.059]
	4 Years	0.989 [0.678, 1.325]	1.068 [0.856, 1.287]	0.193 [0.120, 0.267]	1.806 [1.506, 2.119]

Abbreviations: ACS, acute coronary syndrome. Data indicate absolute reduction in the cumulative incidence of events (percent [95% CI]) derived from linear mixed models employing 1000 bootstrap samples as described in Methods. Time is years from index angiography.

© 2023 Kovach CP et al. *JAMA Network Open.*

Supplemental Figure 1. Flow Diagram for the Formation of the Analytic Cohort.

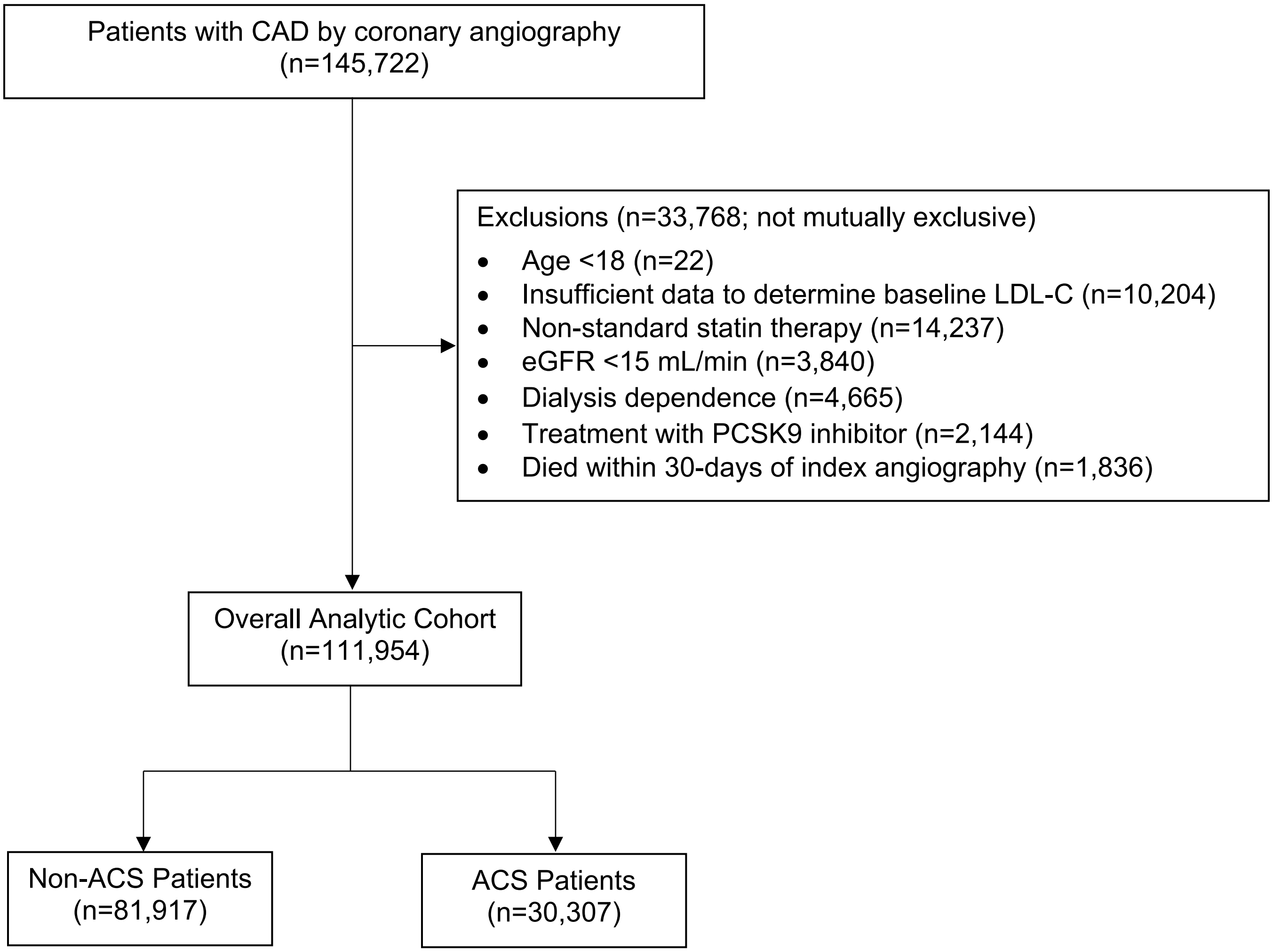

© 2023 Kovach CP et al. *JAMA Network Open.*

Supplemental Figure 2. Estimated Cumulative Incidence of Adverse Outcomes with Observed and Optimized Lipid-Lowering Therapy.

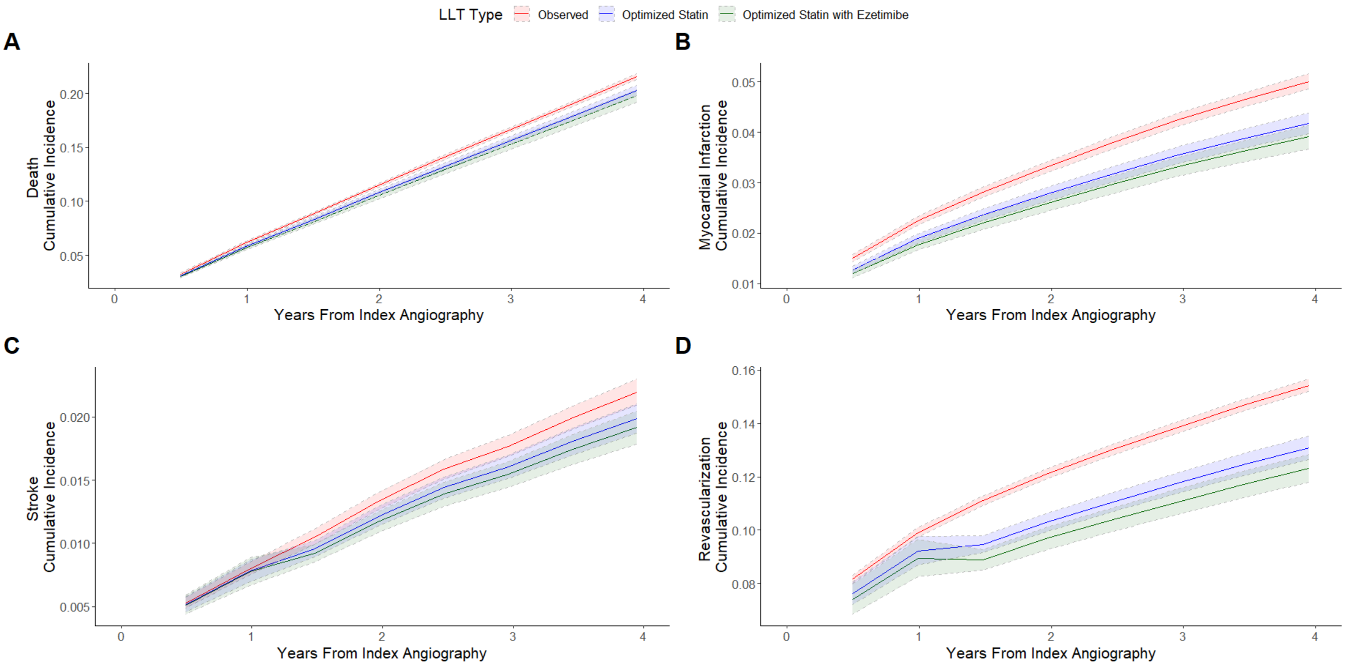

Estimated cumulative incidences of death (A), rehospitalization for myocardial infarction (B), rehospitalization for stroke (C), and coronary revascularization (D) with observed LLT, optimized LLT with statin, and optimized LLT with statin and ezetemibe.

© 2023 Kovach CP et al. *JAMA Network Open.*
