# Supplementary material for: Projected Outcomes of Optimized Statin and Ezetimibe Therapy in US Military Veterans with Coronary Artery Disease
Source: JAMA Netw Open. 2023 Aug 28;6(8):e2329066. doi: 10.1001/jamanetworkopen.2023.29066 (PMC10463102; doi:10.1001/jamanetworkopen.2023.29066)
Supplement: Supplement 2. — Data Sharing Statement [file jamanetwopen-e2329066-s002.pdf]

Data Sharing Statement

Kovach. Projected Outcomes of Optimized Statin and Ezetimibe Therapy in US Military Veterans with Coronary Artery Disease. *JAMA Netw Open*. Published August 31, 2023. doi:10.1001/jamanetworkopen.2023.29066

Data
Data available: No

Additional Information
Explanation for why data not available: The data that support the findings of this study may be made available upon reasonable request, though they will be subject to the stringent data privacy rules of the VA Healthcare System and the United States Government.
